# Plastic changes induced by muscle focal vibration: A possible mechanism for long-term motor improvements

**DOI:** 10.3389/fnins.2023.1112232

**Published:** 2023-02-22

**Authors:** Guido M. Filippi, Angelo Rodio, Luigi Fattorini, Mario Faralli, Giampietro Ricci, Vito E. Pettorossi

**Affiliations:** ^1^Fondazione Policlinico Universitario Agostino Gemelli, IRCCS, Rome, Italy; ^2^Department of Neuroscience, Università Cattolica del Sacro Cuore, Rome, Italy; ^3^Department of Human Sciences, Society, and Health, University of Cassino and Southern Lazio, Frosinone, Italy; ^4^Department of Physiology and Pharmacology “V. Erspamer”, Sapienza Università di Roma, Rome, Italy; ^5^Department of Medicine and Surgery, Otorhinolaryngology Section, Università degli Studi di Perugia, Perugia, Italy; ^6^Department of Medicine and Surgery, Human Physiology Section, Università degli Studi di Perugia, Perugia, Italy

**Keywords:** long-term potentiation, physical exercise, rehabilitation, proprioception, motor cortex

## Abstract

Repetitive focal vibrations can induce positive and persistent after-effects. There is still no satisfactory interpretation of the underlying mechanisms. A rationale, which can provide consistency among different results, is highly desirable to guide both the use of the application and future research. To date, interpretive models are formulated to justify the results, depending on the specific protocol adopted. Indeed, protocol parameters, such as stimulus intensity and frequency, intervention time and administration period, are variable among different studies. However, in this article, we have identified features of the protocols that may allow us to suggest a possible common mechanism underlying the effectiveness of focal vibration under different physiologic and pathologic conditions. Since repetitive focal muscle vibration induces powerful and prolonged activation of muscle proprioceptors, we hypothesize that this intense activation generates adaptive synaptic changes along sensory and motor circuits. This may lead to long-term synaptic potentiation in the central network, inducing an enhancement of the learning capability. The plastic event could increase proprioceptive discriminative ability and accuracy of the spatial reference frame and, consequently, improve motor planning and execution for different motor functions and in the presence of different motor dysfunctions. The proposed mechanism may explain the surprising and sometimes particularly rapid improvements in motor execution in healthy and diseased individuals, regardless of specific physical training. This hypothetic mechanism may require experimental evidence and could lead to extend and adapt the application of the “learning without training” paradigms to other functional and recovery needs.

## 1. Introduction

Specific, intensive, long-term physical training is usually required to achieve improvements in motor performance or motor recovery after functional impairment. An ambitious goal is to minimize the required training period or to ameliorate outcomes with non-invasive complementary approaches. Proprioceptive training has been shown to be frequently proposed (Aman et al., 2015) as a feasible procedure to improve motor function, due to the crucial role of proprioceptive signals in motor control (Wiesendanger and Miles, 1982). To ameliorate motor performance, several authors stimulated proprioceptive sensors by applying a prolonged and repeated mechanical vibration on a muscle or tendon (RFV, repeated focal vibration). RFV has been found to induce, in most cases, long-term (days, weeks, or months) improvements of motor performances in both healthy and diseased individuals (Murillo et al., 2014; Souron et al., 2017a; Alghadir et al., 2018; Fattorini et al., 2021). Even though the results were mainly related to the function of the single treated muscle, RFV has been found to induce complex, unexpected, and still unexplained motor effects also involving untreated muscles. Moreover, RFV ameliorates motor performance in diseased individuals even in the presence of opposite motor disabilities, such as muscle weakness and paresis or spasticity on the other (Karnath, 1994; Kerkhoff, 2003; Brunetti et al., 2006, 2012, 2015; Filippi et al., 2009; Pietrangelo et al., 2009; Camerota et al., 2011, 2016, 2017; Celletti et al., 2011, 2015, 2017a; Marconi et al., 2011; Caliandro et al., 2012; Pettorossi and Schieppati, 2014; Pettorossi et al., 2015; Rabini et al., 2015; Pazzaglia et al., 2016; Attanasio et al., 2018; Russo et al., 2019).

Therefore, to provide a possible justification for the focal vibration effectiveness in such variety of effects it is necessary to propose a common mechanism of action in normal and dysfunctional conditions. The basic idea of this proposed mechanism is that a repeated high frequency mechanical stimulation may facilitate synaptic plasticity along the proprioceptive pathway and in central motor area. These changes should be able to increase the responsiveness of the network and its adaptive abilities (Marconi et al., 2008, 2011; Rocchi et al., 2018). This is in analogy with what has already been observed in other sensory systems, where non-specific stimulation of other sensory system has been shown to induce specific and selective learning, enhancing sensory discrimination and motor learning (Pleger et al., 2003; Frenkel et al., 2006).

## 2. New idea for learning along the proprioceptive system

Sensory stimulation, such as repetition of tactile, visual or auditory stimuli, has been shown to improve perceptual resolution (Pleger et al., 2003; Frenkel et al., 2006; Clapp et al., 2012; Beste and Dinse, 2013), persisting over time. This after-effect was attribute to a specific mechanism termed “learning without training,” a “learning induced in simple or complex motor or sensory performance without specific training, with the aim of changing perception and behavior” (cit. Beste and Dinse, 2013). These authors suggest that these effects, elicited by repetitive sensory stimulation, are likely due to the induction of synaptic modification, like Long-term Potentiation (LTP) and Long-term Depression (LTD) mechanisms, along the afferent pathways and in the primary and secondary somatosensory areas. Specifically, with regard to the tactile system, it has been shown that the spatial discrimination threshold is lowered by high-frequency tactile stimulation and it returns to control after low frequency stimulation (Ragert et al., 2008). These behavioral results resembled those induced by high frequency-LTP and low frequency-LTD *in vitro* (Bliss and Lomo, 1973; Stanton and Sejnowski, 1989; Bliss and Collingridge, 1993; Abraham and Williams, 2003). Furthermore, an important role of *N*-methyl-D-aspartate (NMDA) receptors in this synaptic plasticity has been demonstrated in human behavioral studies using memantine, a substance that selectively blocks the NMDA receptors (Dinse et al., 2003). A single dose of memantine was found to suppress learning, both behaviorally and in cortical circuits, providing evidence for the involvement of NMDA receptors in training-independent sensory learning.

In analogy with these findings, we suggest that the high frequency, long-lasting proprioceptive stimulation may elicit learning in the proprioceptive pathway through a change in synaptic activity that increases the input resolution and the learning capability. The proposed RFV-induced adaptive mechanism could guide further researches to analyze central excitability changes and to define the best practices to combine the RFV after-effects with traditional rehabilitation and reconditioning protocols.

To provide evidences for the suggested mechanism, we report the RFV after-effects, observed in healthy and diseased individuals, in section 1. In the section 2, we highlight the characteristics of vibratory stimulation that support the induction of plastic events underlined our proposed mechanisms. Finally, in section 3 we report spinal and cortical nervous circuitry changes that could justify long-lasting RFV after-effects.

## 3. Section 1: Immediate and sustained complex motor improvements induced by RFV

### 3.1. In healthy individuals

Several studies have examined the effects of focal vibration on healthy individuals. In a recent review (Fattorini et al., 2021) the focus was on the long-lasting after-effects of RFV, ranging from 24 h to several months after the end of RFV, in healthy individuals. In most of the articles listed in the review, only one muscle was stimulated. Only occasionally two or more muscles were stimulated in the same experiment. These investigations reported improvements in strength (Pietrangelo et al., 2009; Lapole and Pérot, 2010; Iodice et al., 2011, 2019; Souron et al., 2017b; Feltroni et al., 2018), power (Pietrangelo et al., 2009), fatigue resistance (Fattorini et al., 2006; Casale et al., 2009; Aprile et al., 2016; Feltroni et al., 2018), rate of force development (Fattorini et al., 2006). These effects were detected at the first test after the end of RFV, as early as 24 h (Filippi et al., 2009; Lapole and Pérot, 2010; Brunetti et al., 2012; Contemori et al., 2021), or < 60 min (Iodice et al., 2011; Aprile et al., 2016; Feltroni et al., 2018; Filippi et al., 2020; Contemori et al., 2021). The different authors considered such short latency as an important expression of a direct RFV action on the nervous system. Then, the effects continued with a persisting or increasing trend until the end follow up, lasting days (Casale et al., 2009; Lapole and Pérot, 2010; Iodice et al., 2011), weeks (Fattorini et al., 2006; Aprile et al., 2016; Souron et al., 2017b; Feltroni et al., 2018; Filippi et al., 2020; Contemori et al., 2021), months (Filippi et al., 2009; Pietrangelo et al., 2009; Brunetti et al., 2012). In some of these studies, the same protocol in the same subject produced effects that commonly require different, specific physical training protocols. For example, quadriceps vibration improved both peak power and fatigue endurance (Filippi et al., 2020), or peak velocity and fatigue endurance (Fattorini et al., 2006; Aprile et al., 2016; Filippi et al., 2020), or power and knee laxity (Brunetti et al., 2012).

In addition, much more complex and multi-joint motor functions have been analyzed in other studies. Body balance (Filippi et al., 2009; Brunetti et al., 2015), movement fluidity (Aprile et al., 2016), and accuracy (Contemori et al., 2021), have been found to improve after RFV. Improvements in body balance, particularly under closed-eye conditions, were obtained by stimulating the quadriceps muscle by RFV (Filippi et al., 2009; Brunetti et al., 2015). All these effects cannot be the result of simple re-modeling of restricted nervous circuits since balance involves a multi-modal muscular activation on different body segments to manage the center of body mass. Moreover, smoothness of a multi-joint movement is a parameter that requires precise proprioception-mediated efferent-afferent control for the coordination of multiple muscles in different body segments (Aprile et al., 2016; Contemori et al., 2021). In conclusion all the above studies support the effectiveness of the RFV not only in modulating the local motor responses in the territory of the vibrated muscle but also in interfering with the central circuits controlling posture and movements.

### 3.2. In diseased individuals

Studies are available on subjects with different motor impairments, caused by central neurological diseases, peripheral neuropathies, aging, osteoarthritis, orthopedic problems, consequences of surgery. The same RFV protocol facilitated motor recovery both in the presence of negative signs of motor deficit as well as in asthenia, weakness, paresis, poor body balance, etc., (Brunetti et al., 2006, 2012, 2015; Filippi et al., 2009; Pietrangelo et al., 2009; Celletti et al., 2011, 2015, 2017a,b; Rabini et al., 2015; Pazzaglia et al., 2016; Attanasio et al., 2018), and in the presence of positive signs, spasticity, hypertonia, contractures, etc., (Camerota et al., 2011, 2016, 2017; Marconi et al., 2011; Caliandro et al., 2012; Celletti et al., 2017b; Russo et al., 2019; Toscano et al., 2019). In contrast, in such opposite pathological conditions motor recovery could be commonly achieved by using deeply different and highly specific physical exercises or pharmacological interventions.

This uncommon and, seemingly, paradoxical aspect is associated with other non-obvious findings, highlighted in other studies, which show positive effects involving districts and functions outside of the part treated. RFV could, at least in part, attenuate a local functional deficit and this, in turn, could lead to a development of new and more adequate compensatory strategies (Camerota et al., 2011, 2017; Marconi et al., 2011; Pazzaglia et al., 2016). However, usually, compensatory strategies would develop gradually, with a relatively long time, while improvement of muscular strength and/or power was induced immediately (after 1 or 24 h) in chronic patients (Brunetti et al., 2006; Filippi et al., 2009; Celletti et al., 2011, 2017a; Rabini et al., 2015). Body balance, mainly in closed eye conditions, (Brunetti et al., 2006, 2015; Filippi et al., 2009; Rabini et al., 2015; Attanasio et al., 2018) largely improved. Findings in chronic patients were often detected in the absence of any other physical therapy (Filippi et al., 2009; Celletti et al., 2011, 2015; Brunetti et al., 2015; Rabini et al., 2015; Pazzaglia et al., 2016; Attanasio et al., 2018; Russo et al., 2019). When RFV was integrated with a conventional rehabilitation program, a powerful potentiating effect emerged compared with rehabilitation alone (Brunetti et al., 2006; Marconi et al., 2011; Caliandro et al., 2012; Rabini et al., 2015). Improvements of the balance in static (Brunetti et al., 2006, 2015; Filippi et al., 2009; Celletti et al., 2011, 2015; Rabini et al., 2015; Pazzaglia et al., 2016; Attanasio et al., 2018) and dynamic conditions (Celletti et al., 2011, 2015; Rabini et al., 2015; Pazzaglia et al., 2016; Attanasio et al., 2018) suggested the involvement of untreated muscles and joints.

Unexpected effects have been observed even in the perception of position and movement, both for the subjective straight ahead (Karnath, 1994; Kerkhoff, 2003) and for the velocity of body movement (Pettorossi and Schieppati, 2014; Pettorossi et al., 2015), suggesting that intense proprioceptive activation increases the perception of movement over time and changes the internal arrangement of the spatial reference frame.

## 4. Section 2: Features of protocols for achieving the sensory-motor learning

### 4.1. Proprioceptive activation

The most common characteristics of the RFV used in the studies cited above includes oscillation with vibratory frequency of 100 Hz and application time of at least 10 min. Direct evidence of the effectiveness of these high frequency and duration was provided by different studies, also involving the space perception after RFV (Pettorossi and Schieppati, 2014; Pettorossi et al., 2015). The magnitude and persistence of the effect have been shown to be consistently observed using frequencies above 80 Hz and application time more than 8 min (Pettorossi et al., 2015).

Regarding the frequency 100 Hz, it is important to underline that this frequency is an appropriate vibration frequency to stimulate the muscle spindles (Burke et al., 1976; Roll and Vedel, 1982) and to evoke the phenomenon of “spindle driving,” i.e., Ia afferent discharge is driven at the same stimulation frequency (Bianconi and Van Der Meulen, 1963). Thus, RFV at 100 Hz may drive the Ia afferent discharge at the same frequency. Interestingly, such a frequency is often used to induce plastic reorganization of central nerve networks, *in vitro* (Bliss and Lomo, 1973; Stanton and Sejnowski, 1989; Bliss and Collingridge, 1993; Abraham and Williams, 2003) and *in vivo* (Iriki et al., 1989) by means of an elevated glutamate synaptic release. This can lead to synaptic events such as LTP and result in an immediate and sustained change in synaptic responsiveness, followed, later, by sustained reorganization of the synaptic pathway.

Regarding the duration, it is well-known that prolonged stimulation allows activation of transcription and transduction of proteins that influence genomic expression at the nuclear level. The activation of these mechanisms could explain the persistence of the effect over time (Lynch, 2004). *In vitro* and *in vivo* studies show that stimulation (or training) must be repeated and organized in a spaced training, i.e., over days, to allow optimal memory consolidation, which is superior to that achieved by a massive training. Similarly, the consequence of intense sensory stimulation requires repetition over consecutive days to ensure long persistence of the training-independent sensory learning (Smolen et al., 2016).

In conclusion, the similarities between the characteristics of vibratory and *in vitro* stimulation suggest that the effects of vibratory protocols may be fundamentally due to an LTP-like mechanism that can develop plastic reorganization in the CNS.

### 4.2. Status of the vibrated muscle

Another aspect to note concerns muscle status during vibration. In the reported studies showing complex and sudden after-effects, the vibration was applied while the subject maintained the vibrated muscle in a state of mild voluntary isometric contraction. In the absence of this condition, the results were contradictory, showing either short (Fattorini et al., 2021) or no effects (Brunetti et al., 2006, 2012, 2015; Fattorini et al., 2006; Marconi et al., 2008; Filippi et al., 2009; Pettorossi et al., 2015).

The apparently more effective results of RFV, applied on contracted muscle, seem to support the mechanism of synaptic learning. Vibration coupled with voluntary activation of nerve circuits, involved in the control of the treated muscle (expressed by muscle contraction), could be a typical model of the Hebbian paradigm for inducing plastic changes in the central nervous system (Marconi et al., 2008, 2011; Pettorossi and Schieppati, 2014; Pettorossi et al., 2015). However, we cannot rule out that vibration effectiveness is due to the stimulus power absorption by the biological system. Indeed, the stimulus transmission is modulated by mechanical coupling and proprioceptive response. The former is related to the muscular stiffness i.e., fibers recruitment and their length, promoting the transmission of vibrations by reducing input damping and distortion (Fattorini et al., 2016, 2017). The latter concerns the proprioceptive local modulation managed by γ-drive able to regulate the spindle sensitivity (Burke et al., 1976).

## 5. Section 3: Evidence of long-lasting excitability changes induced by RFV in the nervous circuits

Several studies have investigated the effects of muscle vibration on nerve circuits, exploring some parameters of spinal and cortical excitability. Spinal excitability is affected by muscle vibration, as shown by depression of the H-reflex after vibratory stimulation (Souron et al., 2017a; Rocchi et al., 2018). These effects have been attributed to a decrease in motoneuron excitability by corticospinal electrical stimulation studies (Souron et al., 2019). In addition, intense vibratory stimulation has been found to induce both a decrease in spinal excitability and a cortical excitability modulation revealed by comparing cortical *versus* thoracic or cervico-medullary evoked potentials (Kennouche et al., 2022; Pfenninger et al., 2023). It should be noted that the duration of these spinal and cortical changes has not been evaluated because the above studies were conducted under acute conditions. However, other authors showed that H reflex and reciprocal spinal inhibition, after RFV, returned to baseline within 60 min (Rocchi et al., 2018).

As with spinal tests, several studies have reported changes in cortical excitability after muscle vibration (Souron et al., 2017a). Acute effects, after a single session of FV, are equivocal regarding cortical excitability, showing evidence of potentiation, decrease, or absence of effects in magnetic evoked potentials (MEPs), short intracortical inhibition (SICI), intracortical facilitation (ICF) (Rosenkranz and Rothwell, 2006; Souron et al., 2017a; Kennouche et al., 2022; Pfenninger et al., 2023). The reason for these discrepancies may be due to differences in the frequency, amplitude, duration, and application modes of vibration (Rosenkranz and Rothwell, 2006). Conversely, studies on chronic, long-lasting effects of RFV on the cortical excitability, are few (Marconi et al., 2008, 2011). In these studies, on magnetic stimulation of primary motor cortex, before and after RFV, Marconi and co-workers obtained long-lasting effects in both healthy (Marconi et al., 2008), and diseased subjects (Marconi et al., 2011), showing a time course consistent with that described in section 1 (2–3 weeks, the duration of follow-up). Furthermore, in the second work (Marconi et al., 2011) neurophysiological tests were correlated with recovery of motor functions. Interestingly, these researchers applied RFV protocol in tune with the parameters described in section 2 and used by the studies mentioned in section 1. Authors, in both the studies, evidenced a that short intracortical inhibition (SICI) changed persistently increasing in the treated muscles, and decreasing in the untreated antagonist. Such a rebalance, between agonist-antagonist muscles, suggests a specific and simultaneous up- and down-regulation of intracortical GABAergic circuits (Marconi et al., 2008, 2011). In these experiments, intracortical facilitation, motor threshold and H-reflex were tested. Intracortical facilitation did not change, and the motor threshold decreased only in post-stroke patients (Marconi et al., 2011), remaining unchanged in healthy subjects (Marconi et al., 2008). H-reflex, tested 1 h after RFV ending, was unchanged both in healthy and diseased individuals. This is not in contrast with Rocchi et al. (2018), since they showed that the H-reflex returned to the baseline within 60 min after RFV ending. Finally, in post-stroke patients, a significant and parallel correlation was shown among SICI increase, threshold reduction and motor improvements. The authors suggested that cortical changes could allow more efficient and selective muscle activation, associated with an improvement in the role of the antagonist, which can reduce the mechanical impedance of the joint during the movement (Marconi et al., 2008, 2011). A current view is that a SICI increase could lead to highly coordinative abilities (Stinear and Byblow, 2003; Dai et al., 2016; Mouthon and Taube, 2019), in healthy and diseased individuals, to avoid the possible development of unwanted co-contractions and dyssynergia, which could be an obstacle to movement (Liepert et al., 2000; Stinear and Byblow, 2003; Marconi et al., 2011; Dai et al., 2016; Mouthon and Taube, 2019).

**FIGURE 1:**
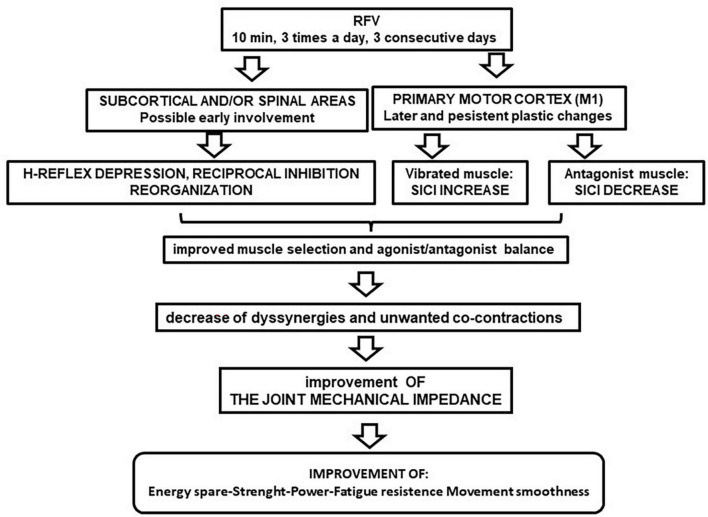
Flow chart of repeated focal vibration (RFV) after-effects on nervous excitability. The suggested cascade of functional implications is illustrated.

A possible schematic sequence of these plastic changes, after RFV application, is shown in Figure 1. The cortex evidenced persistent rearrangements, that are temporally coherent with the behavioral results reported in section 1, whereas the spinal excitability showed only transient change. Therefore, the spinal cord excitability change does not seem relevant for long-term effects, even if it could initially contribute to facilitate the induction of the persistent cortical plastic events.

## 6. Discussion

**FIGURE 2:**
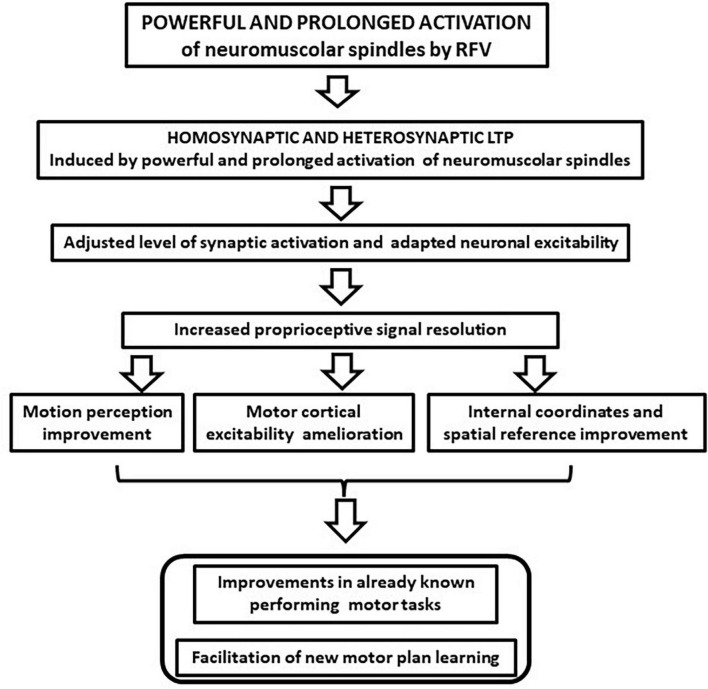
The proposed repeated focal vibration (RFV) synaptic changes and the reported effects on the sensory system. The possible increase of the motion perception and the improvement of the internal and external reference system might favor motor planning and execution.

In this article, we argue that the motor improvements induced by RFV could find a possible theoretical explanation in the ability of non-specific sensory stimulation to induce long-term hetero- and homo-synaptic effects in the CNS, such as LTP, and subsequent neural plastic rearrangements (Beste and Dinse, 2013). This possibility was observed in the tactile, visual, and acoustic sensory systems where unspecific repetitive stimulation induced better discrimination of inputs (Pleger et al., 2003; Frenkel et al., 2006; Clapp et al., 2012; Beste and Dinse, 2013). Beste and Dinse (2013) referred to this mechanism as “learning without training,” to emphasize how the effects were not related to the stimulus specificity. It is conceivable that similar learning can be observed in the proprioceptive sensory system following RFV. This can be considered a form of proprioceptive training (Aman et al., 2015) that can induce both a local increase of sensory discrimination and a central neural plasticity, synergistically improving motor performance (flow chart of the effects is depicted in Figure 2). We pointed out that RFV may generate synaptic potentiation in the proprioceptive circuits and can shift the activity of neural circuits to a different level to enable the system to be more responsive and adaptive. Evidence to support this adaptive mechanism of action is based on certain features of the vibration stimulation protocol that are decisive in achieving functional benefits. The first piece of evidence is the need to use high frequency vibratory stimulation. In fact, positive effects have been observed only after the application of vibration frequencies above 80 Hz, up to 300 Hz, mostly 100 Hz (Rosenkranz and Rothwell, 2004, 2012; Pietrangelo et al., 2009; Iodice et al., 2011; Pettorossi and Schieppati, 2014; Pettorossi et al., 2015). Other authors reported that it is possible to induce positive and persistent after-effects with prolonged and repeated stimulation and these are relevant parameters for inducing plastic processes (Smolen et al., 2016). Furthermore, the effectiveness of RFV increases when the subject pays attention to the area where the vibration is applied or it is associated with the contraction of the activated muscle (Rosenkranz and Rothwell, 2012; Pettorossi et al., 2015). This further supports the possible induction of heterosynaptic LTP, as occurs in Hebbian synaptic plastic rearrangements (Hebb, 1949; Marconi et al., 2008, 2011).

Several studies have shown significant improvements in balance control, suggesting that RFV increases proprioceptive circuit resolution. In fact, the improvement in balance control under closed-eye conditions (Filippi et al., 2009; Celletti et al., 2011, 2015; Brunetti et al., 2012, 2015; Rabini et al., 2015; Attanasio et al., 2018) and the changes in motion perception (Karnath, 1994; Pettorossi and Schieppati, 2014; Pettorossi et al., 2015) seem reasonably due to an enhanced ability of proprioceptive signals to perceive position and motion. This could also be promoted by a refinement of whole-body position and motion representation in space, in relation to spatial coordinates (Karnath, 1994; Pettorossi and Schieppati, 2014; Pettorossi et al., 2015; Contemori et al., 2021). These sensorial ameliorations might synergically act with central motor changes. On the other hand, intracortical excitability shows the presence of different modulation of SICI relative to agonist and antagonist muscles (Marconi et al., 2008, 2011). Consequently, joint stabilization can be more efficient, by adapting the joint impedance to functional demands. In fact, appropriate modulation of joint impedance can improve muscle strength, power, resistance to fatigue, as well as fluidity and precision of movement. Fine-tuning of joint impedance is critical for the accuracy of motor execution and motor learning. In addition, effective rebalancing of joint impedance, even if localized to a few joints, can be instrumental in improving complex, multi-joint motor tasks, such as balance control and gate, and in refining motor accuracy (Aprile et al., 2016; Contemori et al., 2021). Finally, the ability to optimize functional joint stabilization and impedance is a key determinant of motor learning ability.

In conclusion, it seems evident that the shared features of stimulation protocols, proposed by the studies cited above, could develop motor learning independent of training, which potentially opens up new applicative possibilities. The present view suggests that appropriate proprioceptive stimulation procedures may provide new perspectives, which can improve, and develop motor strategies even in complex motor tasks, in which the proprioceptive modality is engaged.

It is to note that listed RFV studies involve mostly protocols adopting vibration frequency set at 100 Hz. New researches are needed to verify the effective role of protocol characteristics in the presence of different functional and pathological conditions and in combination with specific rehabilitation training. These studies should consider the fact that vibratory stimulation should be intense and reiterate and should exploit the combined activation of different signals to facilitate synaptic potentiation through an hebbian mechanism. Furthermore, new experiments are needed to directly confirm the neural changes elicited by high-frequency stimulation, possibly using strategies able to interfere with the induction of short- and long-term synaptic changes.

## Data availability statement

The original contributions presented in this study are included in the article/supplementary material, further inquiries can be directed to the corresponding author.

## Ethics statement

Ethical review and approval was not required for the study on human participants in accordance with the local legislation and institutional requirements. Written informed consent for participation was not required for this study in accordance with the national legislation and the institutional requirements.

## Author contributions

GF, AR, LF, and VEP conceived the outline of the review. MF and GR supervised project and provided suggestions to the manuscript revisions. All authors read and approved the final version of the manuscript and agree with the order of the presentation of the authors.
